# Initial failure morphology of shallow loess containing millimeter-scale cracks: Insights from CT 3d imaging

**DOI:** 10.1371/journal.pone.0359832

**Published:** 2026-11-05

**Authors:** Luqing Zhao, Cuiying Zhou, Zhen Liu, Qingyi Mu, Penghui Ma, Jianbing Peng

**Affiliations:** 1 Institute of Disaster Prevention and Reduction of the Guangdong-Hong Kong-Macao Greater Bay Area, Guangdong University of Technology, Guangzhou, China; 2 School of Civil and Transportation Engineering, Guangdong University of Technology, Guangzhou, Guangdong, China; 3 School of Geological Engineering and Geomatics, Chang'an University, Xi'an, Shaanxi, China; Guizhou University, CHINA

## Abstract

Frequent shallow loess slope failures in loess regions pose significant threats to both linear infrastructure and local communities. Millimeter-scale initial cracks induced by water, temperature, and weathering are key factors controlling such loess failures. Early diagnosis and prevention remain challenging, largely because the disaster chain triggered by millimeter-scale initial cracks is not fully understood, and the early failure behavior of these cracks is poorly characterized. In this study, laboratory experiments were conducted on loess specimens containing artificial millimeter-scale initial cracks to simulate natural cracks. Uniaxial compression tests combined with CT scanning were performed on specimens with initial cracks of varying characteristics. Three-dimensional structural models of the specimens at initial failure were reconstructed using machine learning methods, and the morphology and structural characteristics of the cracks were analyzed qualitatively and quantitatively. The test results show that the cracks generated in the initial failure state mainly involve wing cracks and extension cracks from the initial crack edges as well as wing cracks extending from both sides, initial cracks of varying characteristics produce different types of secondary cracks. Compared to intact specimens, samples with shallow, 45°-inclined, or shallow-surface compound cracks reached initial failure at substantially lower axial stresses—reduced by 78%, 65%, and 81%, respectively. This reduction is attributed to the development of larger-volume failure cracks in specimens with initial cracks. The distribution, inclination, and combination of millimeter-scale initial cracks exacerbate initial failure. Specifically, deeper initial cracks promote more large-volume failure cracks; 45° inclined initial cracks generate more large-volume failure cracks than horizontal or vertical ones; and two initial cracks produce more large-volume, large-diameter failure cracks than a single crack.

## Introduction

Frequent shallow failures in loess slopes pose a significant threat to the safety of linear infrastructure such as roads and bridges, as well as to pedestrians [1–4]. Millimeter-scale initial cracks, which develop in response to changes in the external environment, are a key structural factor contributing to these shallow loess slope failures [3,5]. Research indicates that such cracks reduce soil strength and thereby compromise the stability of shallow slope layers. The failure process in soil containing these initial cracks typically involves stages of crack initiation, propagation, and ultimate failure [6–10]. The state of initial failure represents a critical precursor to full soil collapse. Consequently, analyzing the initial failure morphology of loess with millimeter-scale cracks provides a fundamental basis for understanding the subsequent chain of disaster processes in the shallow layers of loess slopes [3,11–13].

The initial failure behavior of millimeter-scale cracks under hydrological and mechanical loading has been widely studied [3,14–17]. Under wetting conditions, for example, [18] demonstrated that water infiltration increases the self-weight of overlying soil, generating tensile stresses that widen existing cracks and promote downward propagation, thereby accelerating landslide development. [19] revealed that desiccation-induced volumetric shrinkage creates a heterogeneous tensile stress field within the soil matrix; when these stresses exceed interparticle cohesion, soil particles separate and pre-existing cracks expand. [20] further showed that frost heave during freeze–thaw cycles alter soil particle arrangement, forming a loose, high-porosity structure that facilitates crack propagation. [21] investigated the shear behavior of 3D-printed rock structural planes reinforced by basalt fiber low-carbon geopolymer mortar, finding that the 3D-printed structural planes better reflect actual crack patterns, and that numerous cementitious particles accumulate in the interfacial transition zone to form a superior bonding structure. [22] conducted large-scale model tests on retrogressive slopes failure to investigate the relationship between crack development and the mechanisms of soil multiple retrogressive failures. [23] further reveal that the existence of structural planes changes the stress field in loess slopes, creating preferential yield zones around structural planes, resulting in structural planes becoming more likely to become potential slip surfaces. [24] conducted triaxial creep tests on cracked loess specimens and found that the deformation characteristics of these specimens can be classified into axial deformation, bending deformation and torsional deformation. The deformation characteristics of the specimens change with varying crack angles.

Under mechanical loading, new cracks typically initiate from pre-existing cracks and subsequently propagate and penetrate, leading to the failure of rock or soil masses [25–27]. Early theoretical work by [28] related material strength to crack parameters through an energy-based criterion. More recently, [29] developed a micromechanical damage model that captures wing-crack growth and associated irreversible deformations. Experimental advances include the use of acoustic emission to identify crack initiation thresholds in concrete [30], and digital image correlation (DIC) combined with discrete element modeling to characterize crack morphology in rocks [31]. [32] introduced a novel modeling approach that accounts for the closed state of fractures, simulating the failure of rock specimens with pre-existing cracks under uniaxial compression.

While existing research has extensively examined how millimeter-scale cracks influence strength and deformation during initial failure under load [24–27,33,34], the specific initial failure state of soil containing such cracks under load has received limited attention, particularly the influence of crack characteristics (e.g., location, orientation, and configuration) on the initial failure mode. Soil failure is a continuous dynamic process, a detailed study of its initial state contributes to a deeper understanding of the entire failure process and mechanism. Furthermore, this provides a scientific basis for the early identification of emerging shallow loess disasters.

This study performed uniaxial compression tests integrated with CT scanning on loess specimens containing artificial cracks with varying characteristics (depth, inclination, and configuration). Three-dimensional models of the failure structures were reconstructed using machine learning techniques, facilitating a detailed quantitative analysis of crack evolution at the initial failure stage. These findings provide a visual illustration of the failure evolution process of cracked loess and contribute to a deeper understanding of the failure mechanisms in shallow loess from a formation perspective.

## Research content and methods

### Test subject

The study area is located in Jingyang County, Shaanxi Province, within the southern part of China's Loess Plateau, where the surficial loess deposits are predominantly eolian in origin. The southern Jingyang Plateau spans approximately 70 km^2^, with elevations ranging from 420 to 490 m above sea level and exhibiting a general east-to-west topographic decline. Field surveys show that millimeter-scale cracks are widely developed in the shallow layers of loess slopes (Fig 1a). These cracks separate the near-surface soil into discrete, unstable blocks that are prone to detachment and collapse under external disturbances, contributing to shallow geohazards such as localized slope failure. The cracks are mainly confined to the upper 3 m of the slope surface, with widths typically between 0.1 and 3 mm (Fig 1c), which aligns with the systematic survey results of [5] on the distribution and geometry of millimeter-scale cracks across the Loess Plateau. In shallow loess slopes, such cracks develop at different depths, show variable inclinations, and occur as adjacent pairs in localized soils. Given the stress-unloaded condition of shallow slope loess, the three principal stresses differ markedly in magnitude. This stress state is commonly approximated as uniaxial compression in both research and engineering practice. Accordingly, soil samples were collected from the slope surface for uniaxial compression testing. The intact Q_3_ loess was cut into blocks measuring 400 mm × 200 mm × 200 mm (Fig 1c). These blocks were individually sealed in plastic film to maintain their natural state, with their in-situ orientation clearly marked, and transported to the laboratory in wooden boxes lined with cushioning material.

**Fig 1:**
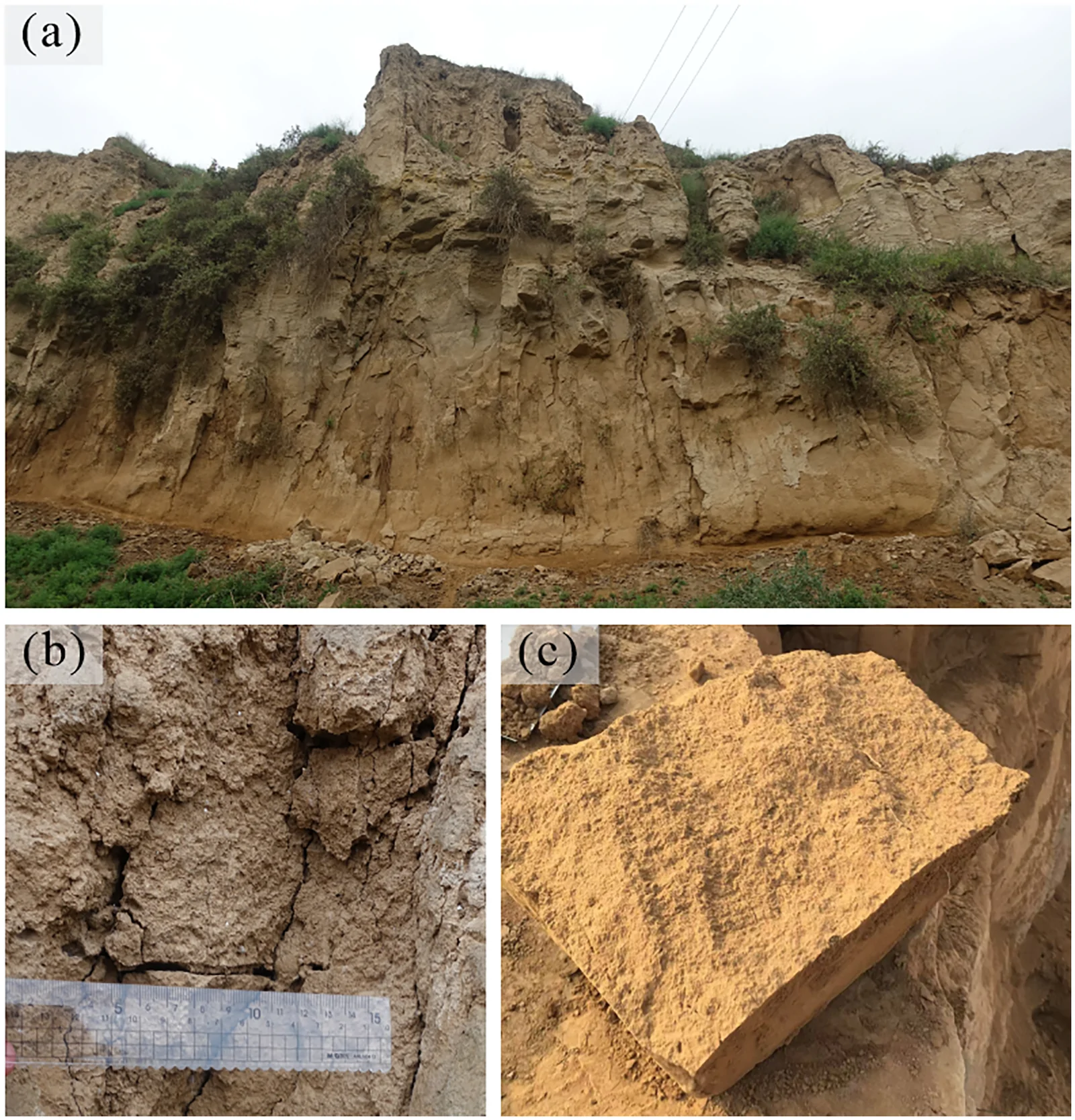
Field surveys and samples collection. (a) Shallow loess slope disaster. (b) Millimeter-scale cracks in shallow loess. (c) Original loess block samples.

The basic physical properties of the intact soil sample are summarized in Table 1, including specific gravity (2.71), dry density (1.4 g cm^-3^), natural water content (14%), void ratio (0.93), liquid limit (30.26%), plastic limit (18.6%), and plasticity index (11.66). The particle size distribution (Fig 2) comprises sand (0.075–4.75 mm, 0.65%), silt (0.005–0.075 mm, 75.38%), and clay (≤0.005 mm, 23.97%). Based on ASTM D 2487‑11 (2011), the intact loess is classified as a low‑plasticity clay (CL).

**Table 1:** Physical properties of loess samples.

Specific gravity Gs	Dry density *ρ*_d_ (g/cm^3^)	Natural moisture content *w* (%)	Porosity ratio *e*	Liquid limit *w*_L_ (%)	Plastic limit *w*_P_ (%)
2.71	1.4	14	0.93	30.26	18.6

**Fig 2:**
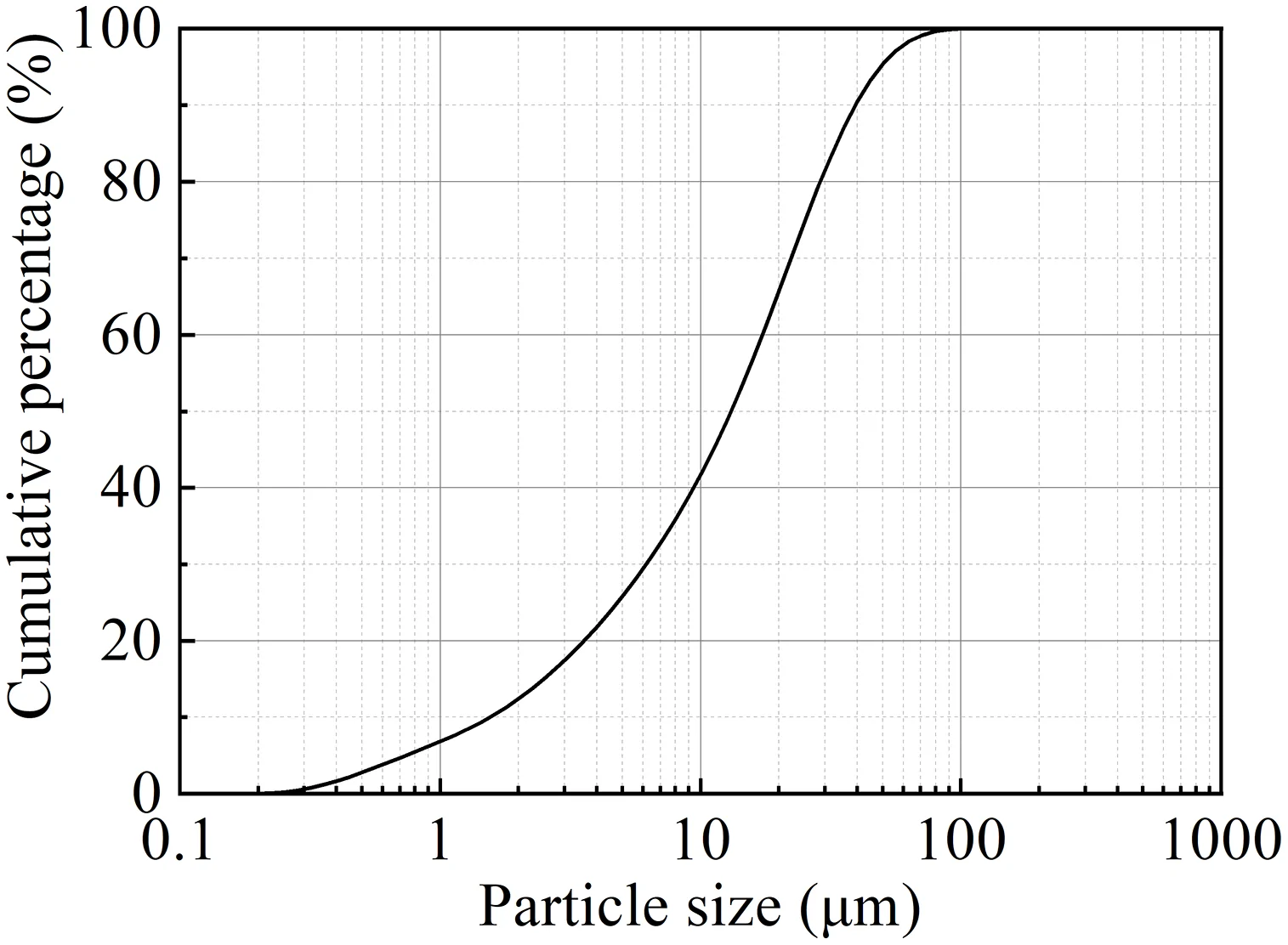
Particle size distribution of loess samples.

### Experiments

Uniaxial compression tests were performed using a YYW-2 strain-controlled unconfined compression apparatus. A custom-designed CT-compatible holder, fabricated from transparent acrylic, was employed to enable in situ X-ray computed tomography (CT) scanning during deformation. This device enables non-destructive observation of internal damage in specimens restrained under defined deformation. The holder incorporates a precision alignment groove at its base, ensuring that specimens at different deformation stages are consistently positioned for CT imaging. This design permits continuous analysis of the deformation process from an identical viewing perspective throughout the experiment.

Loading followed the Standard for Geotechnical Test Methods: axial stress and displacement were recorded at intervals of 0.5% axial strain until 3% strain was reached, and thereafter at 1% intervals. The test was terminated after applying an additional 3–5% axial strain once the axial stress reached a peak or stabilized value. The loading stage at which the first visually crack is detected on the specimen surface can be identified as the initial failure stage [35,36]. The stress-strain curve for this stage shows a deviation from that of the previous elastic stage. [35] used DIC to record time-dependent strains on the specimen surface during loading and found that the variance D increased when microcracks appeared on the specimen surface. The variance D was used to identify initial irreversible damage state of mudstone. In this study, as stress and displacement were recorded at specified strain intervals, the selected initial failure stress should be regarded as an approximate operational value rather than a precise material threshold. Its uncertainty is bounded by the adjacent loading intervals. Once the initial failure state was reached, the specimen was secured in a CT-compatible fixture and immediately transferred for CT scanning.

CT scanning was performed using a nanoVoxel-2000 micro-CT scanner. This system features a high-performance, enclosed X-ray source and a sample chamber capable of accommodating large specimens while supporting multiple acquisition modes, such as helical, offset, and finite-angle scanning. This device can achieve the highest pixel resolution of 0.05 μm and a spatial resolution of 0.05 μm. CT scans were performed on loess specimens in their intact state, initial failure state, and post-failure state. The CT images obtained featured a resolution of 72 μm. The scanning procedure was initiated once axial load and displacement readings indicated the onset of deformation and visual inspection confirmed the presence of surface microcracks. At this point, the specimen within its CT-compatible holder was immediately transferred to the scanner’s sample chamber for imaging.

Following pre-reconstruction processing and enhancement calibration of the acquired CT images, three-dimensional reconstructions were generated using Dragonfly (Comet Technologies Canada, Canada). Quantitative analysis of the reconstructed loess samples was subsequently performed through image segmentation and processing algorithms [37–39]. A schematic overview of the test procedure is presented in Fig 3.

**Fig 3:**
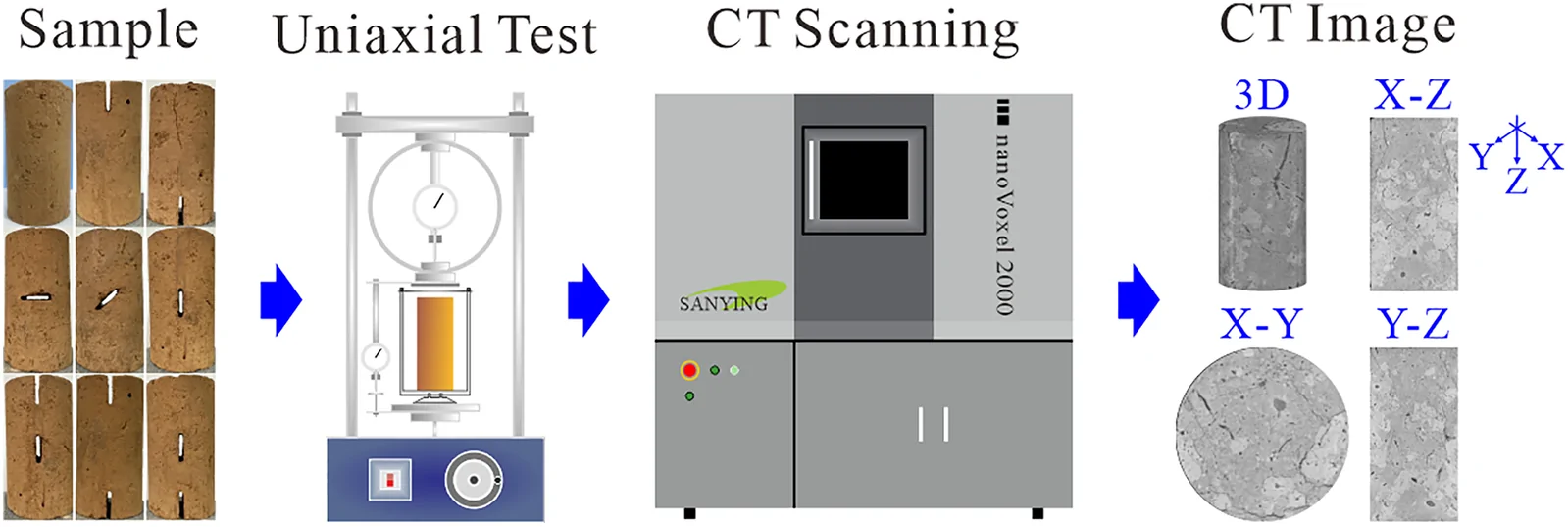
Test procedure.

### Test methods

Based on the distribution and geometric characteristics of millimeter-scale cracks observed in shallow loess, specimens containing cracks with varying characteristics were prepared. A suitable soil cube was first cut from the intact block sample, from which a cylindrical specimen (50 mm diameter × 100 mm height) was trimmed using a wire saw. A transparent PVC sleeve, pre-fabricated with a specified crack pattern template, was employed. The cylindrical loess specimen was placed into this sleeve, and artificial cracks matching the template were introduced using specialized cutting tools. Based on the frequent crack widths reported by [5] and the results of field investigations, the width of the artificial cracks was set at 2 mm and the length at 20 mm. Natural cracks are locally curved and rough, while artificially cracks are simplified from complex shapes with smooth and straight forms. Fig 4 illustrates the prepared loess specimens containing cracks of different characteristics. The specimens are categorized based on crack location, inclination, and combination as follows: top crack (T), middle crack (M), bottom crack (B), middle crack with 0° inclination (M-0), middle crack with 45° inclination (M-45), middle crack with 90° inclination (M-90), combined top and middle cracks (TM), combined top and bottom cracks (TB), and combined middle and bottom cracks (MB). A specimen without any artificial crack served as the intact reference and was labelled I. Tubular channels extending into the interior are visible on the surfaces of these specimens. These pore channels represent a distinct type of microstructure inherent to the original loess, primarily consisting of features such as root traces and wormholes, which are common in such deposits.

**Fig 4:**
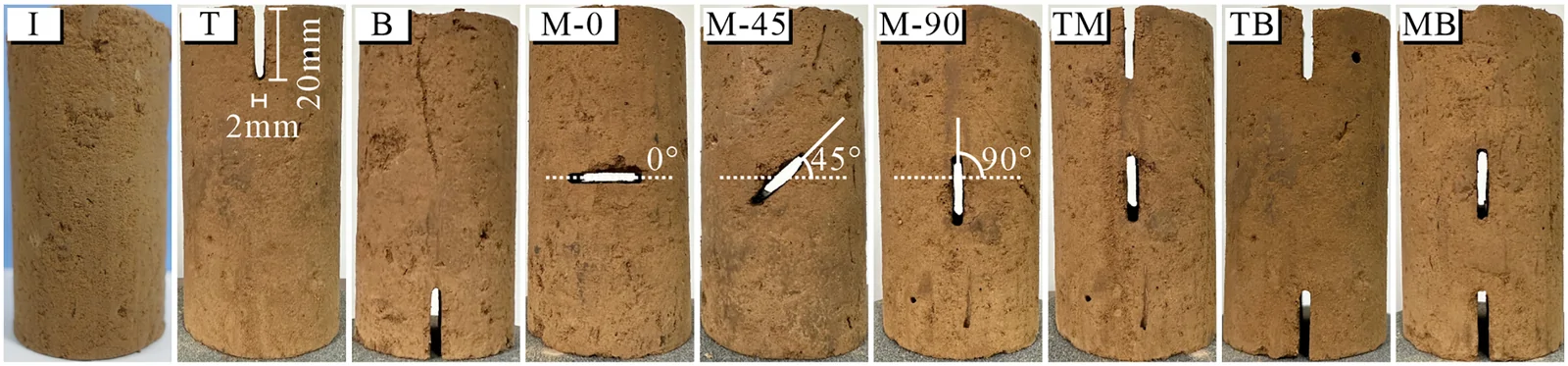
Loess specimens containing millimeter-scale initial cracks.

The loess specimen containing initial cracks, mounted within the CT holder, was placed into the uniaxial compression apparatus for testing. Upon reaching a predetermined strain state during uniaxial compression, the holder maintained this deformation. The holder and specimen were then transferred to the CT scanner sample chamber, where precise positioning was ensured by engaging the base groove with the corresponding alignment feature.

### Data processing

A machine learning approach was employed to identify and extract the internal structure of loess specimens in the initial failure state, ultimately establishing separate three-dimensional models of the soil matrix, cracks, and pores. The training procedure is illustrated in Fig 5. First, a region containing typical loess matrix, cracks, and pores was selected from an X–Y plane CT image. Using threshold segmentation, these components were identified and labeled with distinct colors to create a training dataset. A U-net network, a highly encapsulated AI plugins in the Dragonfly 3D Images software, was applied to these three sample categories to develop a recognition model. The encoder-decoder structure is set to 5 layers, with an initial filter count of 64. The network uses a 2.5D input format, meaning that three consecutive slices are input simultaneously to leverage the spatial contextual information between adjacent slices. Notably, the segmentation results from machine learning directly depend on the segmentation process and may exhibit some deviation from physical reality [40]. Labeled samples were sliced into 64 × 64-pixel training patches with a step size of 16 pixels. The loss function was defined as Ors Dice Loss and optimization was performed using the Adadelta algorithm. After inputting the training data into the model, 100 iterative optimization training steps were conducted. The trained model was then used to recognize the loess matrix, cracks, and pores in arbitrary X–Y plane CT images. Misrecognized results were corrected and incorporated into an updated training set to further refine the model. This process was repeated until the validation index curve stabilized below 0.1, indicating the establishment of a deep learning model capable of reliably distinguishing the three components. This model was subsequently applied to CT images of the entire loess specimen to extract three-dimensional data for cracks and pores.

**Fig 5:**
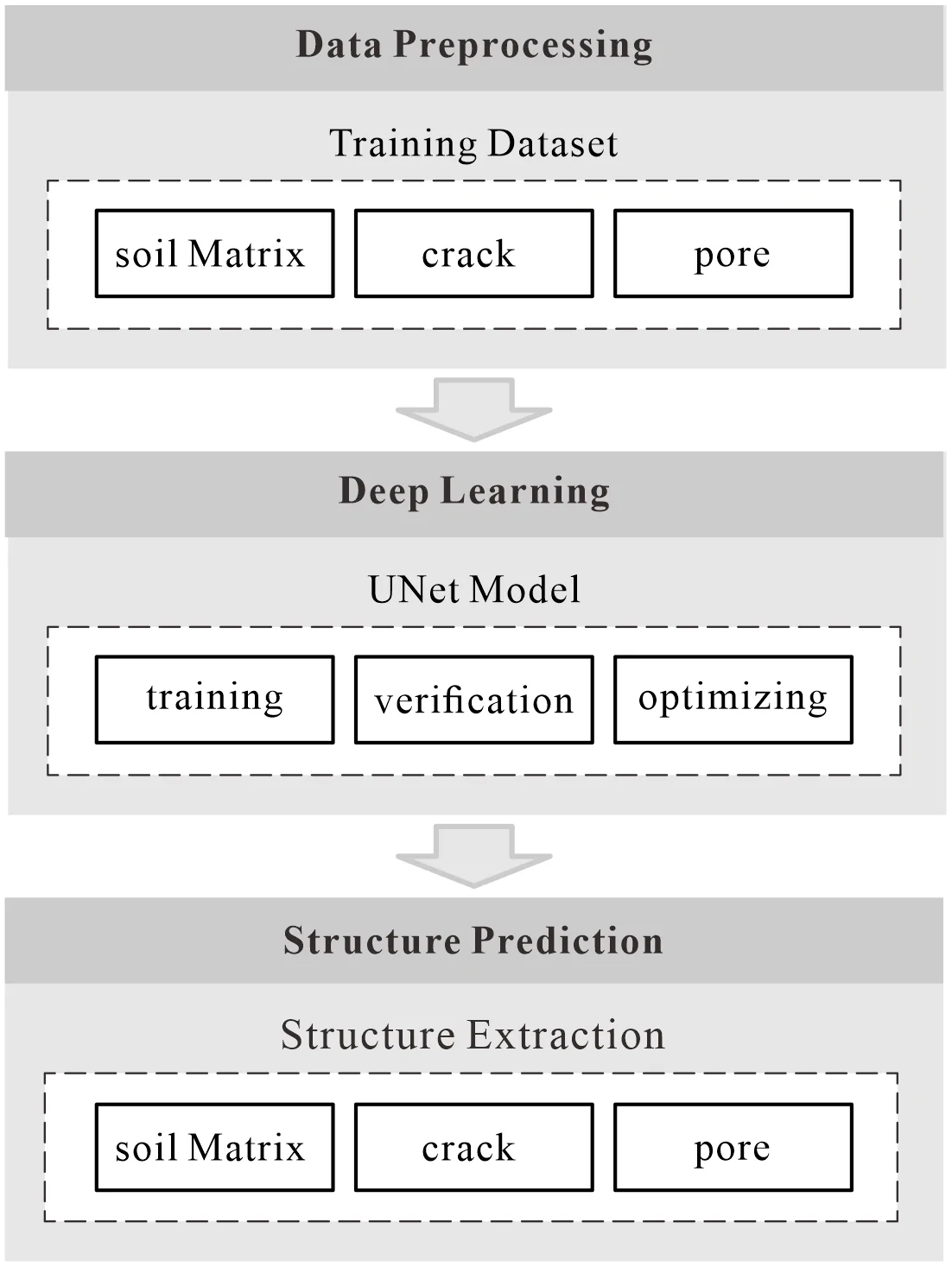
Machine learning methodology process.

The initial failure morphology of the loess samples was analyzed by quantitatively calculating spatial geometric parameters of the cracks and pores. Table 2 presents the geometric parameters of the cracks, including volume, surface area, maximum and minimum Feret diameters, and spatial orientation angles Phi and Theta. Based on the uniaxial compression test results and 3D CT images, the effects of millimeter-scale initial cracks on the initial damage pattern of loess are analyzed from three perspectives: the initial damage stress state, the damage mode, and the geometrical structural characteristics of the cracks.

**Table 2:** Spatial geometric characteristic parameters of cracks.

Characteristic Parameters	Definition
Volume	the volume occupied by the labeled voxels of each discrete object
Surface area	the surface area occupied by the labeled voxels of each discrete object.
Max feret diameter	the longest distance between any two parallel tangents along each discrete object’s convex hull
Min feret diameter	the shortest distance between any two parallel tangents along each discrete object’s convex hull
Phi	the angle between the Z axis and the longest axis of the object, range from 0 to 90 degrees
Theta	the angle between the X axis and the projection of the longest axis of the object on the X-Y plane, range from −180–180 degrees

## Results and discussion

### Initial failure stress state and morphology

Fig 6 shows the uniaxial compressive stress-strain curves of loess specimens with different characteristic initial cracks and their damage processes. The uniaxial compressive stress-strain curves of loess specimens with different characteristic initial cracks show a tendency of increasing and then decreasing. As shown in Fig 6a, specimen I produced an initial failure at an axial pressure of about 65 kPa, and small damage crack was observed on the top of specimen. Upon reaching the damage state, small damage crack expanded and formed the penetration damage crack with an inclination of about 45°. Specimen I was damaged by shear slip along this damaged crack surface. As shown in Fig 6b, specimen T produced initial failure at an axial pressure of about 28 kPa, small secondary wing cracks were observed both lateral to the initial crack at the top and at the bottom of the specimen. Upon reaching the damage state, these lateral wing cracks expanded and penetrated with the initial crack to jointly formed the damage crack surfaces. As shown in Fig 6c, the initial failure of specimen B was caused at an axial pressure of about 16 kPa, and small damage cracks grew at the bottom of the specimen. After reaching the damage state, these cracks expanded and combined with the initial crack at the bottom to form the penetration damage crack surfaces.

**Fig 6:**
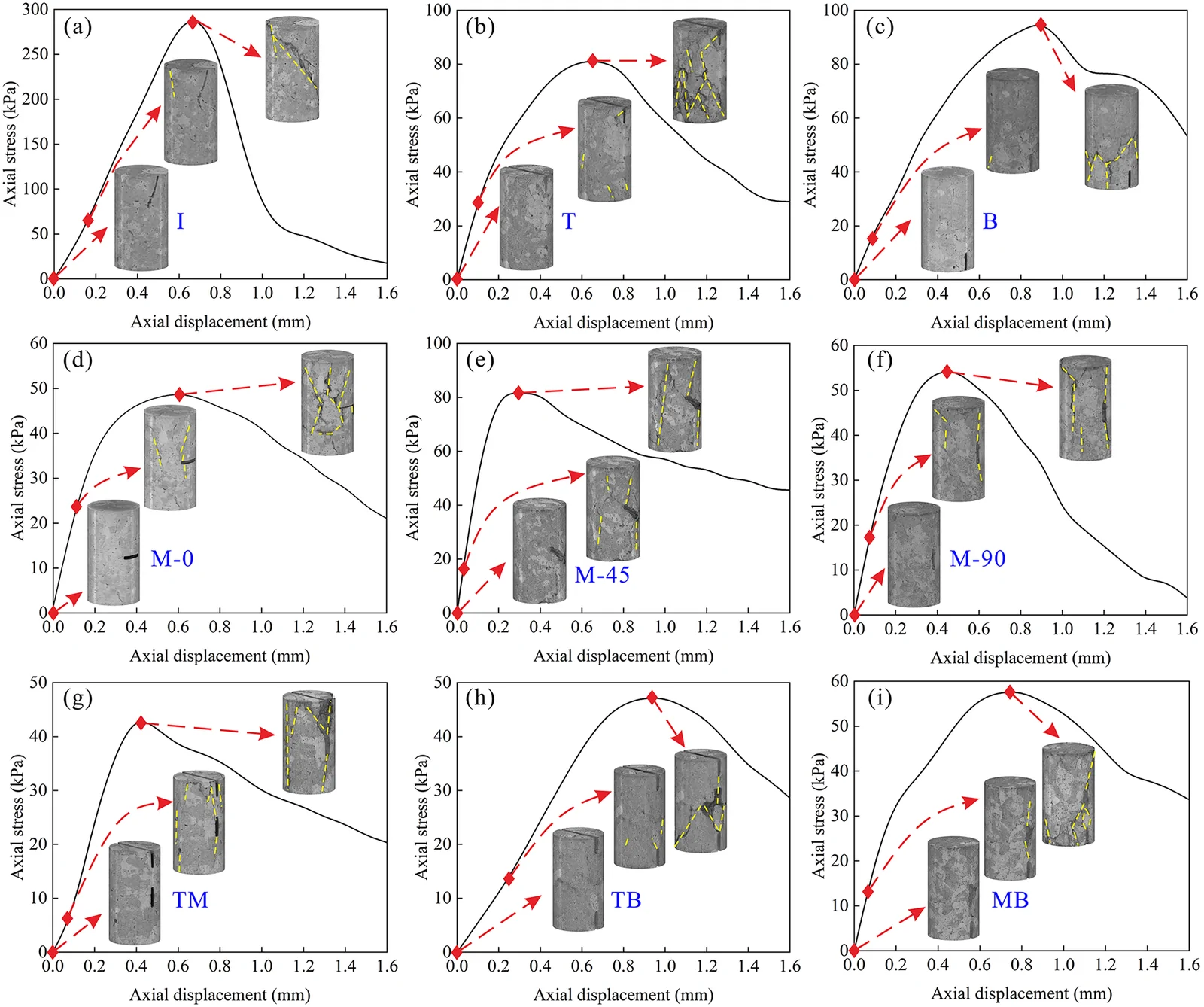
Uniaxial compressive stress-strain curves and damage modes of loess specimen containing initial crack. (a) Specimen I. (b) Specimen T. (c) Specimen B. (d) Specimen M-0. (e) Specimen M-45. (f) Specimen M-90. (g) Specimen TM. (h) Specimen TB. (i) Specimen MB.

For different inclination of initial crack, as shown in Fig 6d, the initial failure of specimen M-0 occurred when the axial pressure was about 24 kPa, the lateral wing cracks grew at both sides of the initial crack with 0° inclination angle, and small damage cracks appeared on the surface of the specimen. After reaching the damage state, the initial crack was closed, and the lateral wing cracks expanded to the top and bottom surfaces of the specimen along its direction, together forming the damage crack surfaces. As shown in Fig 6e, the initial failure of specimen M-45 was generated when the axial pressure reached about 16 kPa, the lateral wing cracks grew at both ends of the initial crack with 45° inclination, and small damage cracks appeared on the surface of specimen. After reaching the damage state, the lateral wing cracks expanded to the top and bottom surfaces of specimen along its direction, and interconnected with other cracks to form the damaged crack surfaces. As shown in Fig 6f, the initial failure of specimen M-90 (M) was caused when the axial pressure reached about 18 kPa, and the extension cracks grew at both ends of the initial cracks with 90° inclination. After reaching the damage state, the edge extension cracks expanded and penetrated through the top and bottom surfaces of specimen, forming the damage crack surfaces together with the initial crack.

For different combination of initial cracks, as shown in Fig 6g, the initial failure of the specimen TM occurred at an axial pressure of about 7 kPa, with the development of wing cracks on the lateral side of the top initial crack and on the two ends of the middle initial crack. After reaching the damage state, these wing cracks expanded and combined with the two initial cracks to form the damage crack surfaces. As shown in Fig 6h, the initial failure of specimen TB happened at an axial pressure of about 13 kPa, and the edge wing cracks grew above the initial cracks at the bottom. After reaching the damage state, these wing cracks expand upward and connect with the initial crack at the top, which jointly form the damage crack surfaces. As shown in Fig 6i, the initial damage of specimen MB occurs when the axial pressure is about 13 kPa, and the edge wing cracks are generated at the upper end of the middle initial crack and the upper end of the bottom initial crack. After reaching the damage state, the edge wing crack at the upper end of the middle initial crack expanded to the upper surface of specimen, and the edge wing crack at the upper end of the bottom initial crack connected to the middle initial crack, and they together formed the damage surface.

Compared to samples without cracks, initial damage developed earlier in samples with initial cracks. Among the specimens containing initial cracks, specimen M is more susceptible to damage than specimen T and specimen B, specimen M-45 is more susceptible to damage than specimen M-0 and specimen M-90, and specimen TM is more susceptible to damage than specimen MB and specimen TB. Initial cracks located in the middle are more vulnerable to cause fast initial damage to specimen, and the 45° inclination angle is more favorable for initial failure in this study.

The internal failure morphology of the loess specimen in the initial damage state is shown in Fig 7. The initial failure cracks of specimen I are basically arranged along the 45° inclination plane, mainly distributed in the top of specimen. It may be related to the device loading pattern. The initial failure cracks of specimen T developed at the lower end of initial crack at top and extended to the bottom of the specimen. The initial failure cracks of specimen B were composed of two parts, one distributed on the side of bottom initial crack and extended upward, another distributed on the top of specimen. The initial failure cracks of specimen M-0 are mainly developed at the two ends and upper side of initial crack and extend inclined upwards. For specimen M-45, one initial failure crack developed at the upper end of initial crack and extended to both ends of specimen, one failure crack extended along the lower end of initial crack at an inclination downward, and the other failure crack extended along the lower side of the initial crack downward. For specimen M-90 (M), one damage crack extended upward and downward diagonally along the lower end of initial crack, and the other extended upward along the upper end of initial crack. For TM specimens, the initial failure crack connected the top initial crack with the middle initial crack and developed upward diagonally along both sides of middle initial crack. In the specimen TB, the initial failure crack extended upward along the right side of initial crack. In the specimen MB, one initial damage crack connected the middle initial crack and the bottom initial crack, and one developed on the side and top of the middle initial crack and extended upwards. Initial damage cracks were generated mainly at the ends and sides of the initial cracks. This is due to the fact that the presence of initial cracks provides structural defects, which are highly susceptible to stress concentrations at both ends and both sides, thus inducing secondary edge crack and secondary wing crack initiation [31,41].

**Fig 7:**
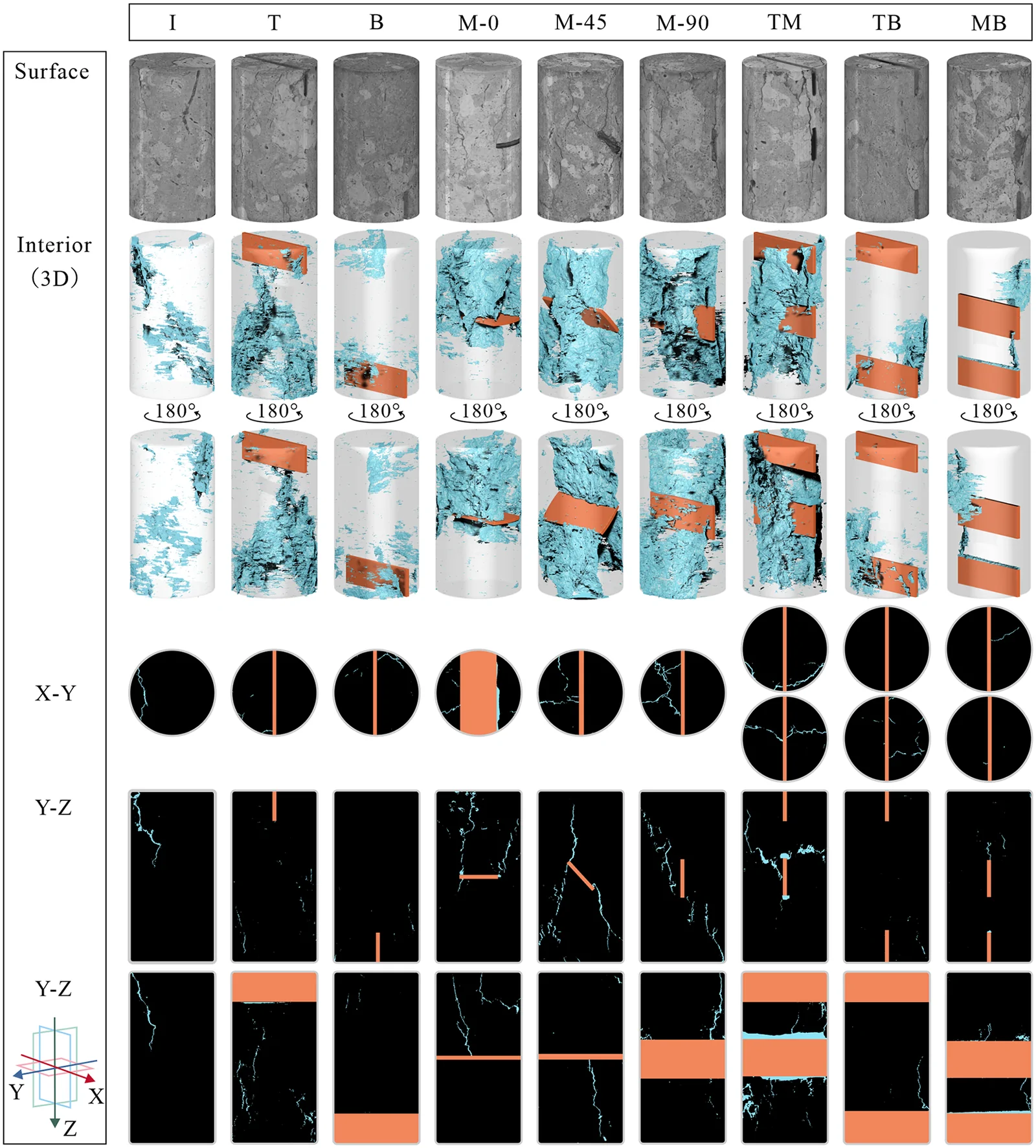
The initial failure crack morphology of loess specimens.

### Structural characteristics of initial failure cracks

Fig 8 shows the volume of failure cracks at the state of initial failure. As shown in Fig 8a, the number of failure cracks in specimen I, specimen T, specimen M and specimen B all increase and then decrease with increasing volume. Compared to Specimen I, specimen T as well as Specimen M and Specimen B resulted in more large volume (1E + 12-1E + 13 μm^3^) failure cracks, with number percentages of approximately 0.02%, 0.02%, and 0.04%, respectively. This is because the initial cracks provide structural defects within the specimen and tend to create stress concentrations around them [12,19,42]. As shown in Fig 8b, the number of failure cracks in both specimen M-0 and specimen M-90 increases and then decreases with increasing volume, and the number of failure cracks in specimen M-45 gradually decreases with increasing volume. At the initial failure state, the 45° initial crack creates more large volume (1E + 12-1E + 13 μm^3^) failure cracks, approximately 0.04%. As shown in Fig 8c, the number of specimen TM, specimen TB, and specimen MB failure cracks all decrease gradually with increasing volume, and the percentage of large volume (1E + 12-1E + 13 μm^3^) fracture cracks is about 0.02%, 0.1%, and 0.07%, respectively. Specimens containing two failure cracks cause more small volume (0-1E + 6 μm^3^) failure cracks and large volume (1E + 12-1E + 13 μm^3^) failure cracks compared to specimens containing no crack or single crack.

**Fig 8:**
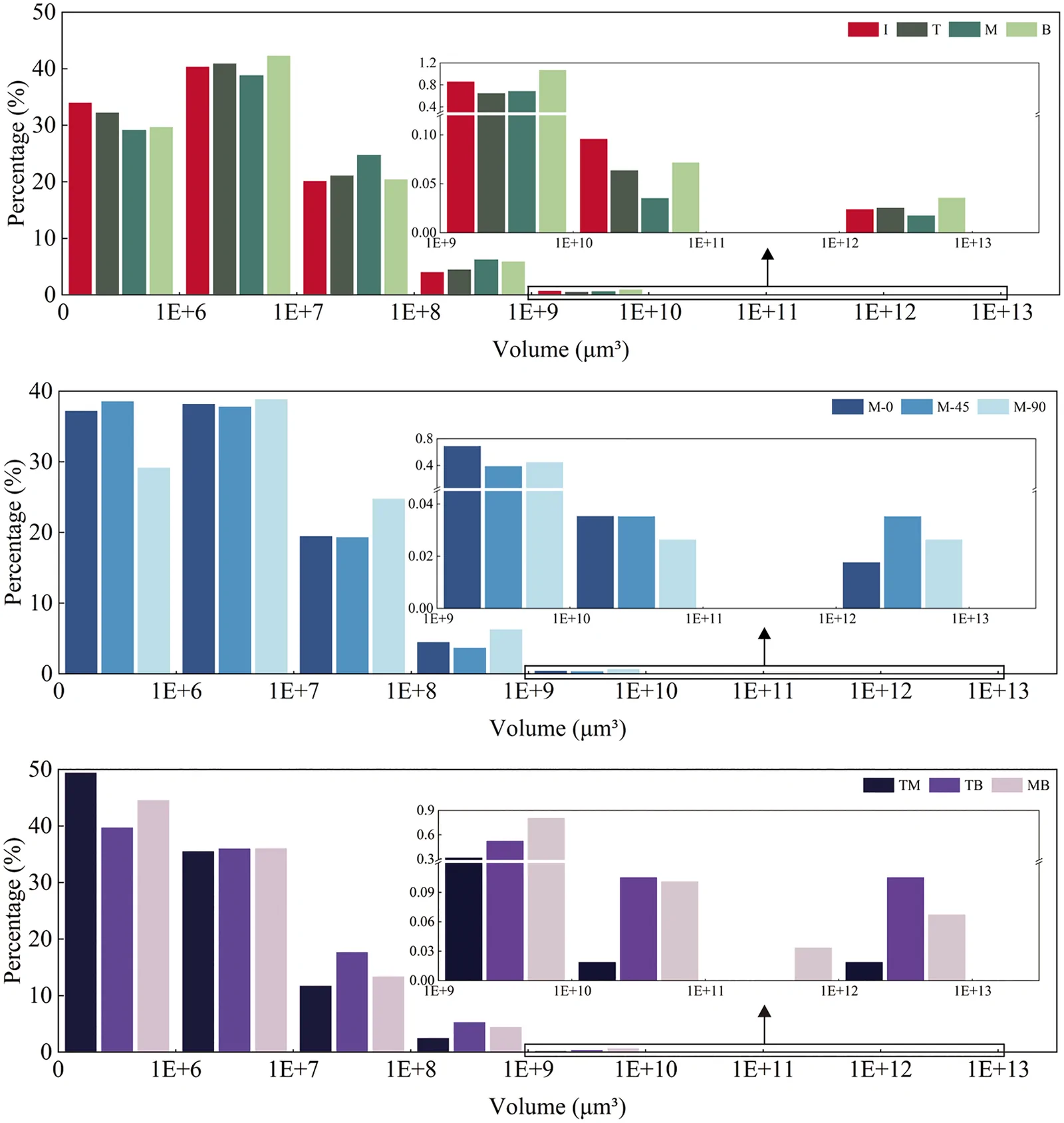
Volume of initial failure cracks. (a) Specimens with different distribution cracks. (b) Specimens with different inclination cracks. (c) Specimens with different combination cracks.

Fig 9 illustrates the surface area of failure crack at initial failure state. As shown in Fig 9a, the number of damaged cracks in specimen I, specimen T, specimen M and specimen B all decrease with increasing surface area, the largest number of cracks are found in the surface area between 0-1E + 6 μm^2^, approximately 89.82%, 89.79%, 86.88% and 88.69%, respectively. Specimen M-0, specimen M-45, specimen TM, specimen TB and specimen MB all show similar laws, the percentage of cracks with surface areas between 0-1E + 6 μm^3^ being approximately 90.21%, 92.47%, 94.73, 89.18% and 91.53% respectively (as seen in Fig 9b, 9c). These also support the above statement.

**Fig 9:**
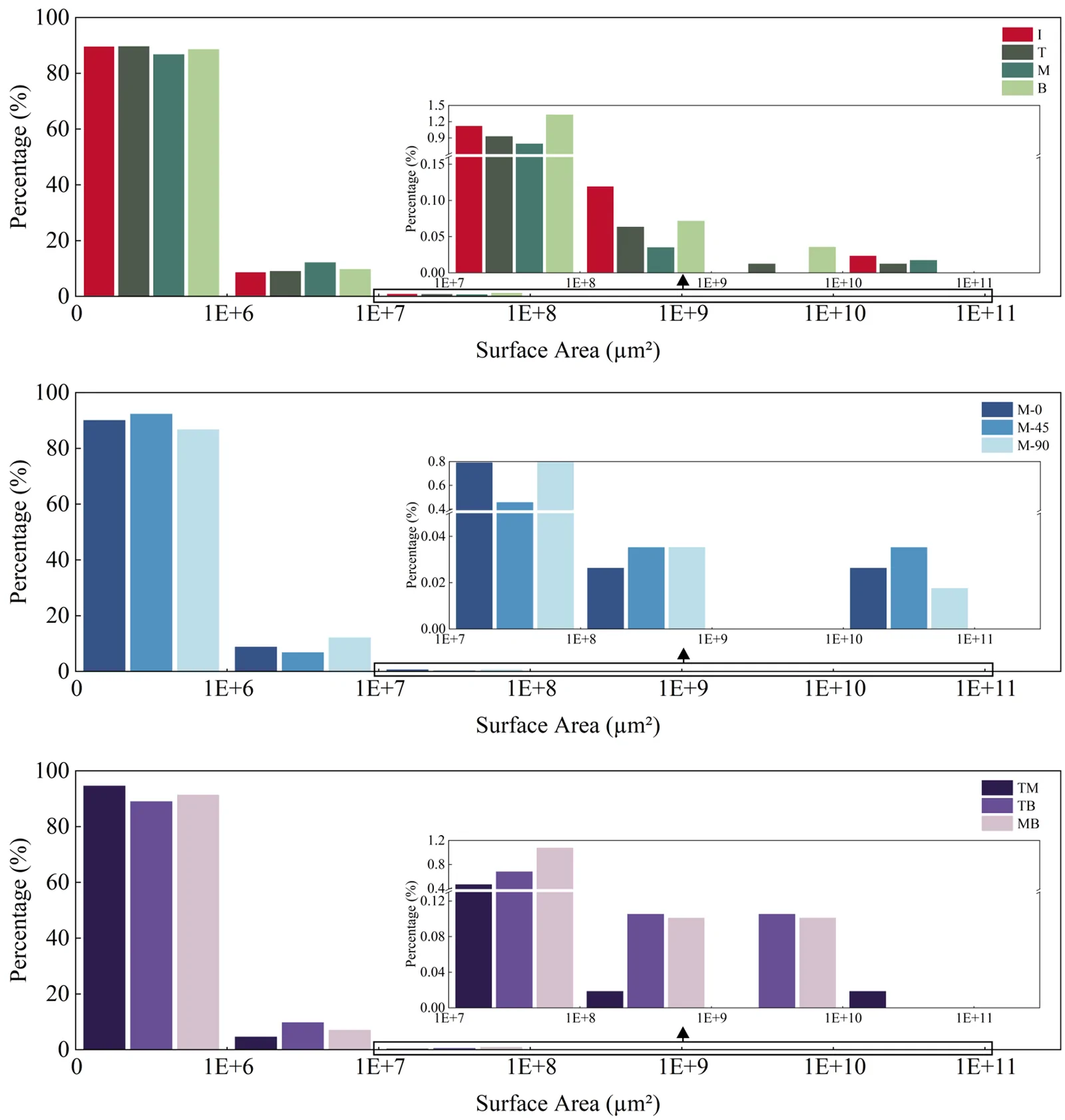
Surface area of initial failure cracks. (a) Specimens with different distribution cracks. (b) Specimens with different inclination cracks. (c) Specimens with different combination cracks.

Fig 10 illustrates the diameter of failure crack at initial failure state. The number of failure cracks decreases with increasing max feret diameter and min feret diameter for all specimens. As shown in Fig 10a, among the specimens containing cracks at different locations, specimen M has the highest percentage of cracks in the max feret diameter range of 1E + 5-1E + 6 μm, approximately 0.02%, specimen T has the highest percentage of cracks in the min feret diameter range of 1E + 5-1E + 6 μm, approximately 0.02%. This indicates that in the initial damage state, the middle initial crack led to thin and long initial failure cracks inside the specimen. As shown in Fig 10b, the number of specimen M-90 failure cracks within the max feret diameter range of 1E + 5-1E + 6 μm accounted for 0.02%, whereas specimen M-0 and specimen M-45 failure cracks show no cracks within this range and the number of the min feret diameters within the range of 1E + 4-1E + 5 μm were larger than that of specimen M-90. As shown in Fig 10c, specimen TM, specimen TB and specimen MB all have a greater number of cracks than a single initial crack specimen in different max and min feret diameter ranges. The specimens containing two initial cracks cause more small failure cracks.

**Fig 10:**
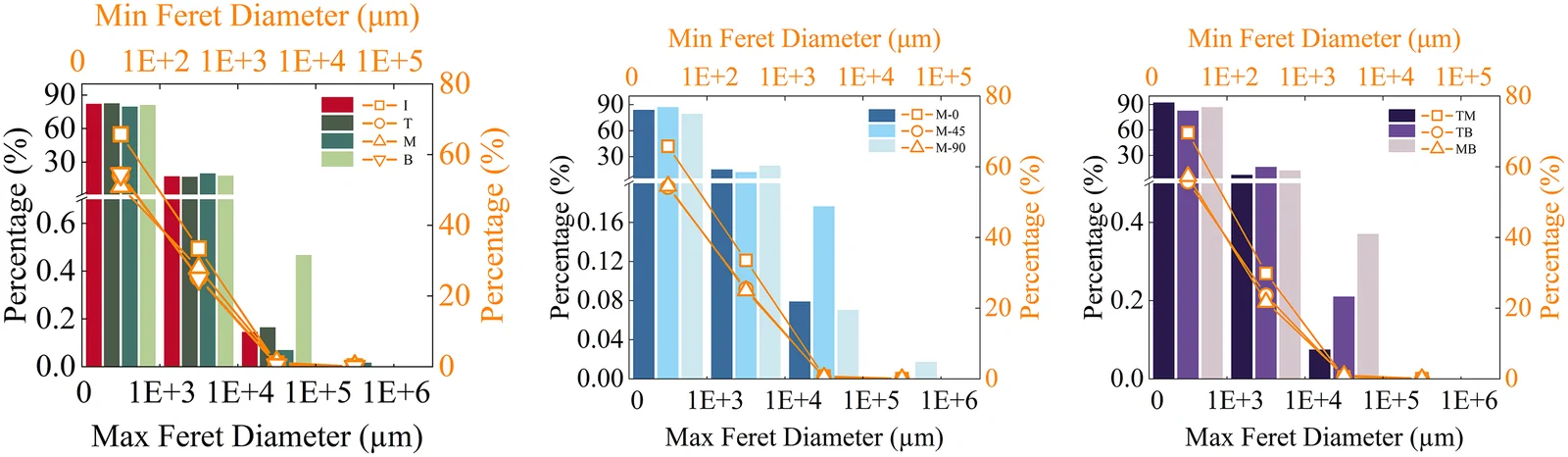
Feret diameter of initial failure cracks. (a) Specimens with different distribution cracks. (b) Specimens with different inclination cracks. (c) Specimens with different combination cracks.

Fig 11 illustrates the spatial inclination Phi of failure crack at initial failure state. The spatial inclination Phi of initial failure cracks in Specimen I is mainly distributed in the range of 75°-90°, approximately 68%. The proximal vertical crack inclination is related to the loading direction. The spatial inclination of the failure cracks of the specimens containing initial cracks have similar pattern, and with the percentage of cracks number with Phi between 75° and 90° in specimen T, B, M-0, M-45, M-90, TM, TB, and MB as 92%, 73%, 90%, 63%, 94%, 83%, 91%, and 55%, respectively. This shows that more failure cracks are nearly vertically distributed in the specimens containing initial cracks, which is related to the spatial inclination of initial crack. Under the action of same direction loading, both ends of the cracks are prone to initiate secondary cracks by extension [19,41]. Specimen M-45 failure cracks are relatively few in number within Phi of 75°-90°, but occupy a larger number in the range of 0–15°, approximately 30%, and specimen N failure cracks have a value of 25% in this range. The reason could be that the 45° initial crack is vulnerable to secondary cracks in the nearly horizontal direction. The specimen TB shares similar patterns with 37% in the range 0–15°. This is because more nearly horizonal failure cracks were generated at the initial failure state (Fig 7).

**Fig 11:**
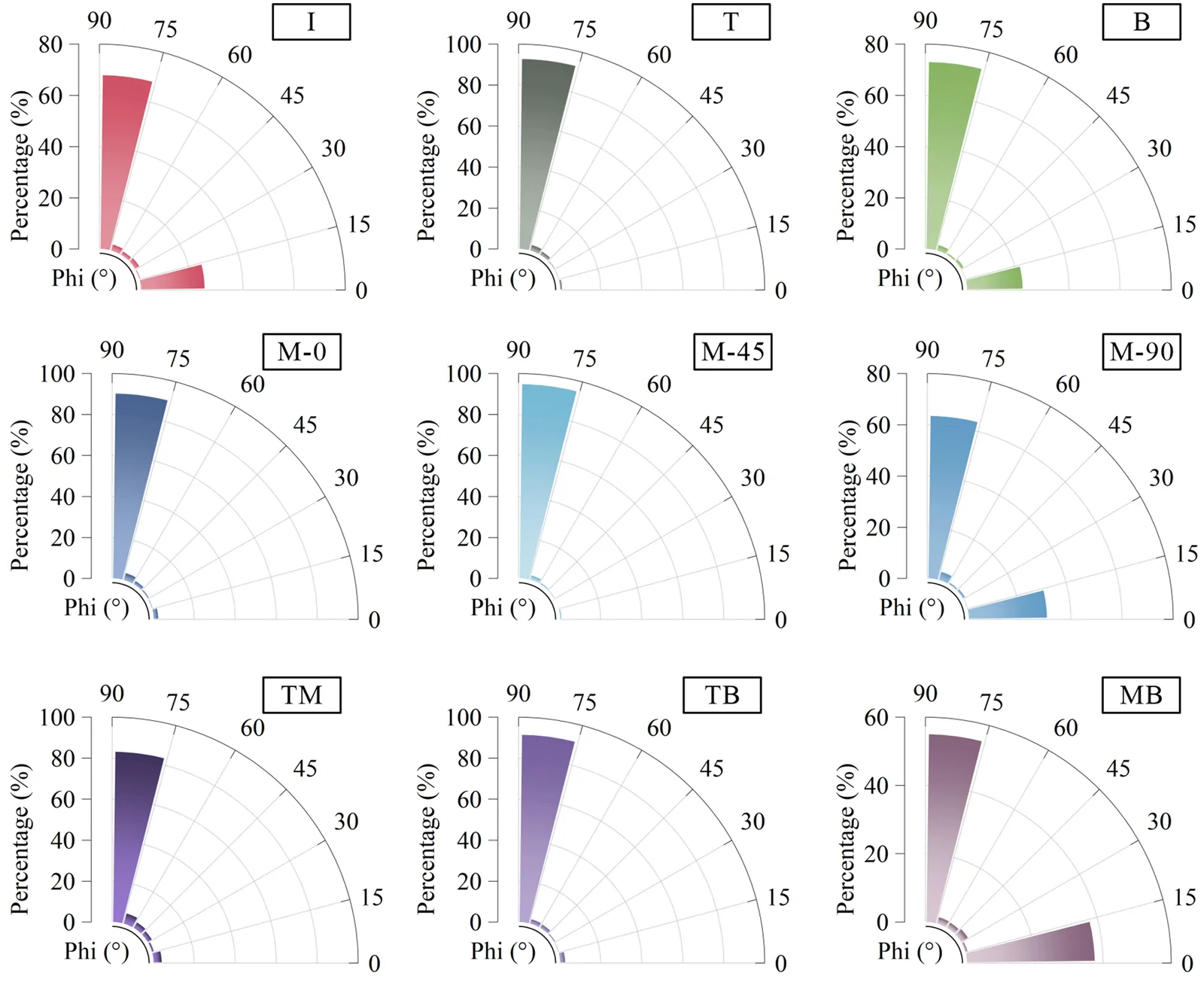
Spatial inclination Phi of initial failure cracks.

Fig 12 illustrates the spatial inclination Theta of failure crack at initial failure state. The spatial inclination Theta of failure cracks in specimen I, B, M-45 and MB are mainly distributed in the range of 180°-210°, approximately 34%, 31%, 39% and 46%, respectively. Meanwhile, spatial inclination Theta of failure cracks in specimen T, M-0, M-90, TM and TB are mainly distributed in the range of 270°-300°, approximately 39%, 50%, 37%, 48% and 42%, respectively. In the initial failure state, the failure cracks are basically arranged along a certain horizontal direction.

**Fig 12:**
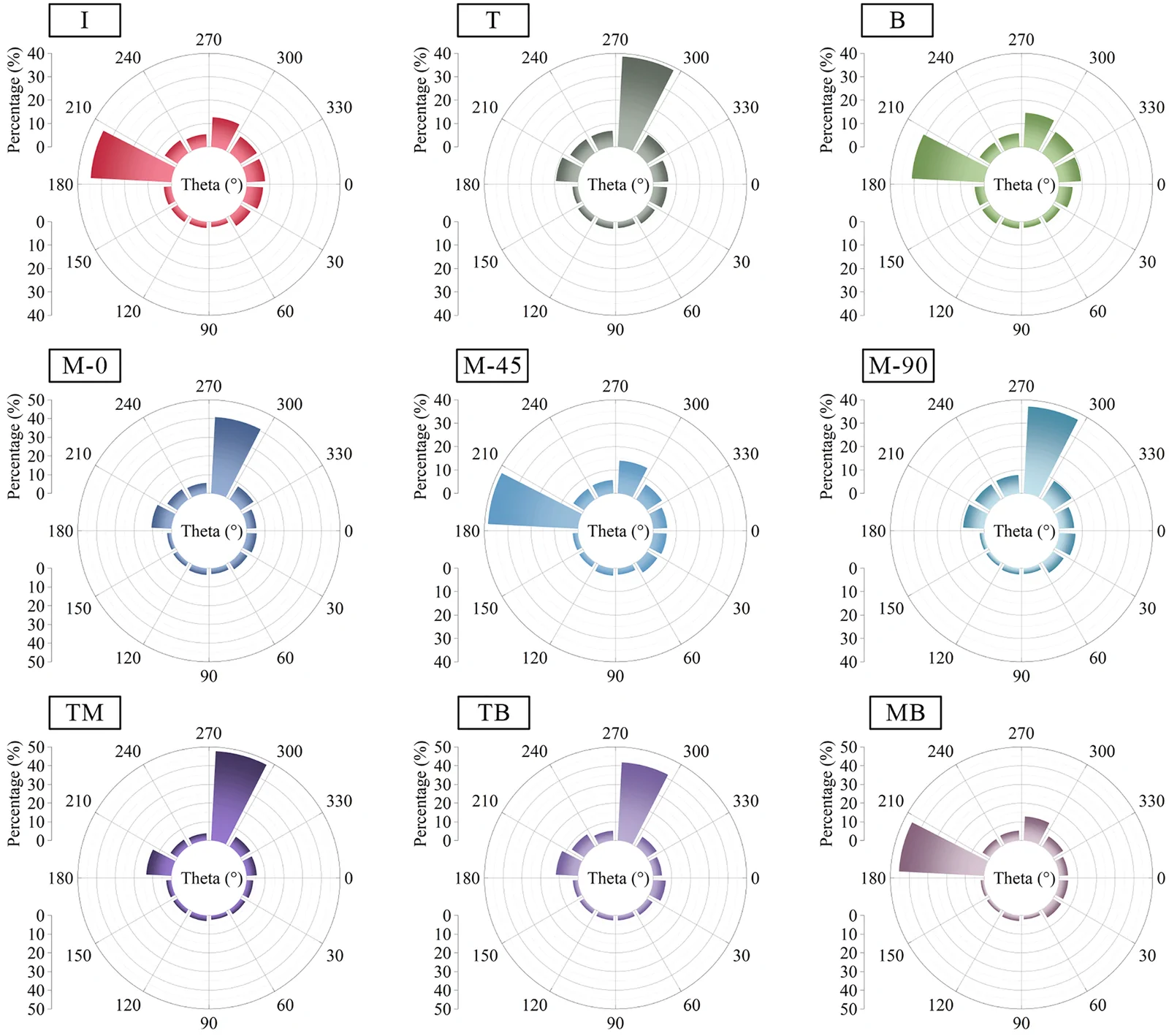
Spatial inclination Theta of initial failure cracks.

## Discussion

The influence of millimeter-scale initial cracks in shallow loess on the initial failure morphology is mainly characterized by the development of secondary failure cracks. To systematically evaluate the initial failure behavior of the specimens containing initial cracks, three typical secondary failure cracks were identified, as shown in Fig 13. The 90° initial crack primarily initiates edge extension cracks, the 0° and 45° initial cracks primarily initiate edge wing cracks and lateral wing cracks (Fig 7). Edge extension cracks initiate at the tip of an existing crack and extend in a path roughly parallel to the direction of the existing crack. Edge wing cracks initiate at the tip of the existing crack, usually initiate in a direction perpendicular to the existing crack, and subsequently extend in the direction of the axial stress. Lateral wing cracks initiate on both sides of the existing crack, usually in a direction perpendicular to the existing crack, and subsequently extend in the axial stress direction to the specimen boundary. [43] discovered similar phenomena. The development of failure cracks is related to existing cracks. Since existing cracks serve as a kind of structural defect, the tip of the crack is prone to form a stress concentration area. Under axial load compression, the crack tip is mainly subjected to tensile stress, shear stress and compound stress. When the stress intensity at the crack tip exceeds the strength of the soil body, the secondary crack grows. Therefore, secondary cracks are often developed along the tip of existing cracks [31,41,44].

**Fig 13:**
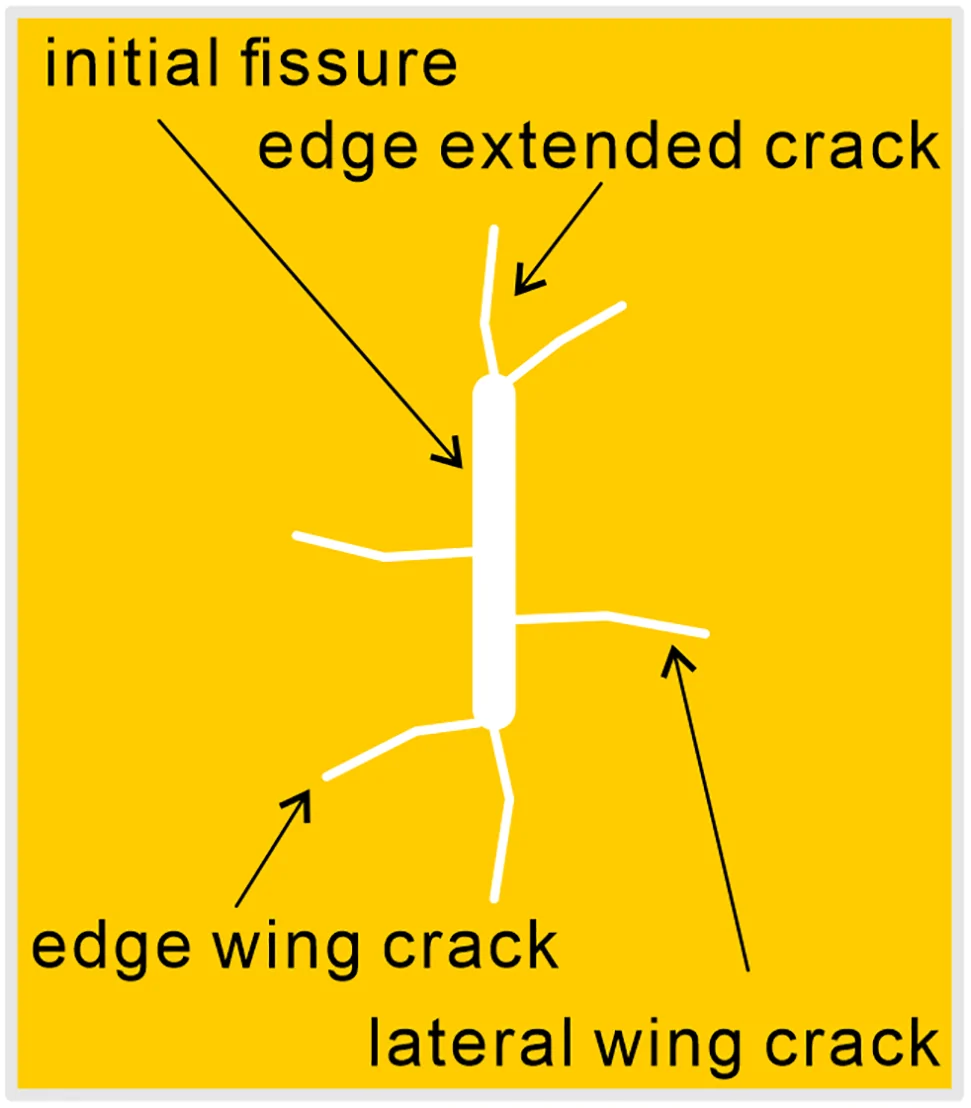
Typical types of secondary cracks.

Scholars first studied the cracking mechanism of geotechnical bodies using the method of fracture mechanics [45,46]. The study of the stress field at the tip of the crack revealed that a small plastic zone exists at the tip of the crack under compression, which is prone to form stress concentrations. The stress field distribution at the crack tip under pressure was analyzed using the real passivated tip crack model proposed by Chenqi [47]. Fig 14 shows the real passivated tip crack model.

**Fig 14:**
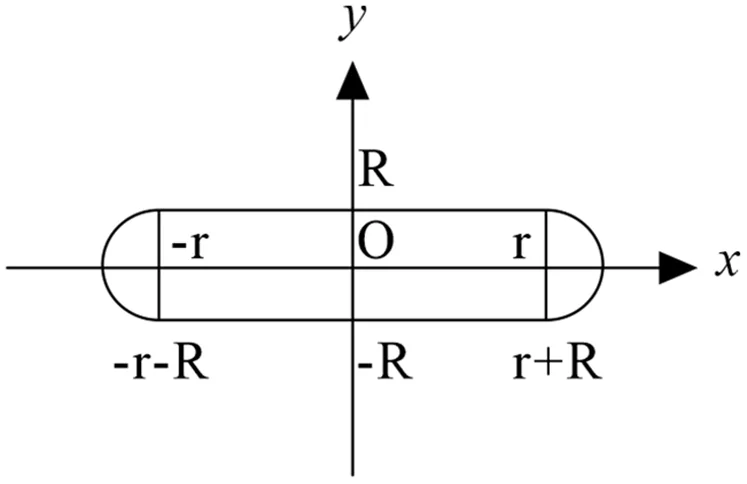
Passivated tip crack model.

The crack-surface equation is calculated as below:


$$\begin{array}{c} \begin{array}{cc} y=\pm R & \left|x\right|\leq r\\ y=\pm \sqrt{R^{\text{2}}-x^{\text{2}}} & r\leq \pm x\leq r+R \end{array} \end{array}$$ (1)

The conformal transformation leading to:


$$\begin{array}{c} z=\omega \left(\zeta \right)=\frac{r}{\text{2}}\left(\zeta +\frac{\text{1}}{\zeta }\right)+R\left(\frac{\zeta }{\text{6}}+\frac{\text{1}}{\zeta }-\frac{\zeta^{\text{3}}}{\text{6}}\right) \end{array}$$ (2)

The tensile stress $\sigma_{\theta }$ around the crack under compression satisfies the following relation:


$$\begin{array}{c} \begin{array}{c} \sigma_{\theta }=\sigma_{\text{1}}\left[\frac{\left(1+\lambda )(3r+R)(r+2R\right)}{Re\left(\omega^{\prime }\left(\zeta_{\text{0}}\right)\right)(3r+7R)}+(1+\lambda )\right]\mathrm{\backslash hfill}\\ -\frac{2(1-\lambda )(3r+R)(r+2R)}{\left(3r+7R\right)}Re\text{\textbackslash{}hspace\{1em}\}\left(\frac{\zeta_{\text{0}}^{\text{2}}e^{-2a}}{\omega^{\prime }\left(\zeta_{\text{0}}\right)}\right)\mathrm{\backslash hfill}\\ +\frac{(1+\lambda )R}{Re\left(\omega^{\prime }\left(\zeta_{\text{0}}\right)\right)}\mathrm{\backslash hfill} \end{array} \end{array}$$ (3)

The numerical solution showed that the maximum tensile stress was mainly concentrated at the crack tip. Under the action of tensile stress, tensile damage occurs at the crack tip, and secondary cracks expand at the tip along the direction of the original crack, forming edge extension cracks [41,48] (Fig 15).

**Fig 15:**
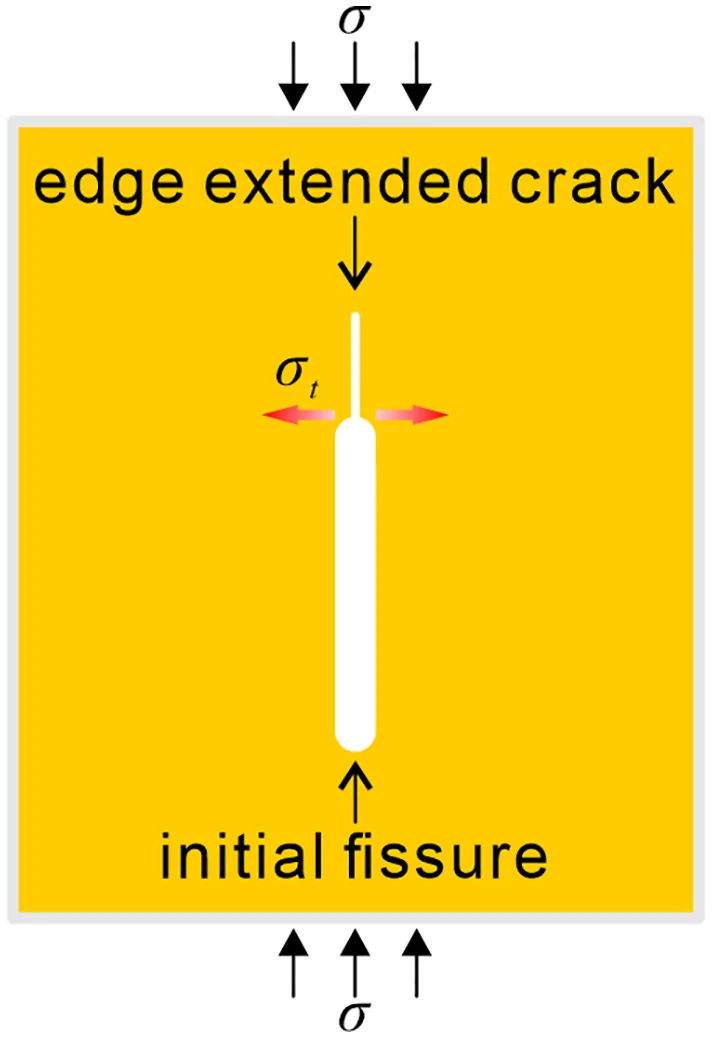
Edge extension cracks under compression.

Loess materials usually behavior brittle damage characteristics. Due to the anisotropic nature of soil structure, the actual stresses on soil body are complex and the crack tips are also subjected to shear stresses. The crack tip stress intensity factor K_II_ calculated under uniaxial compression can be expressed as:


$$\begin{array}{c} K_{II}=\frac{\sigma }{2}\left[\sin2\beta -f\left(1-\cos2\beta \right)\right]\sqrt{\pi a} \end{array}$$ (4)

According to the theory of maximum circumferential tensile stress, the pure type II crack initiation angle is 75°. It can be seen that the direction of the initiation angle of the edge wing crack is parallel or inclined to the direction of the maximum principal stress, i.e., the secondary crack extends along the tip of the existing crack diagonally, as shown in Fig 16. Therefore, 0° and 45° initial cracks tend to generate edge wing cracks most easily.

**Fig 16:**
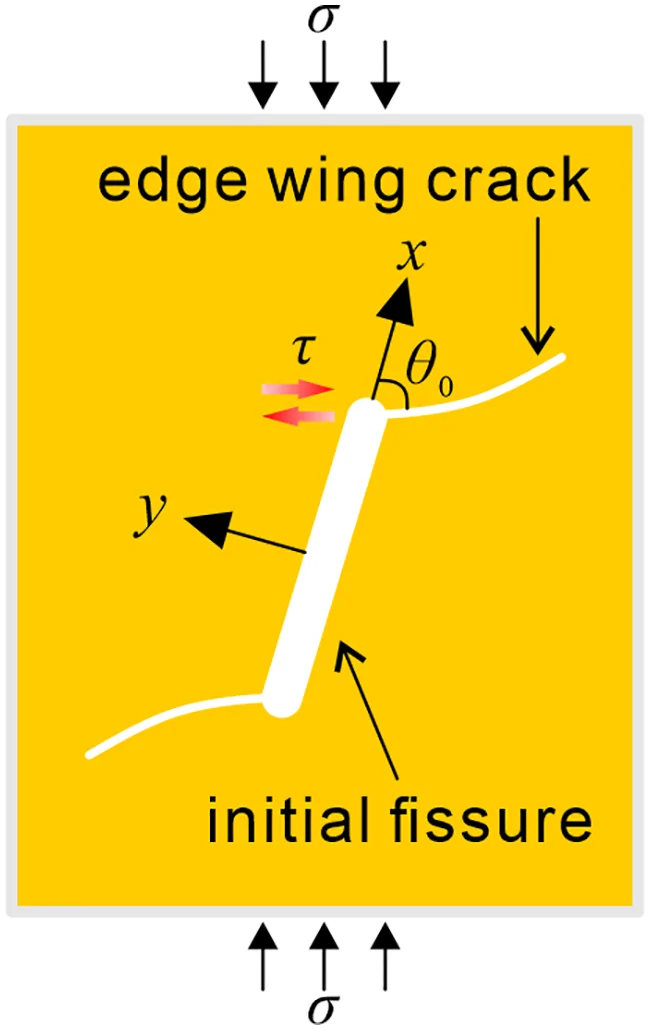
Edge wing cracks under compression.

Lateral wing cracks form on either side of existing cracks and together with existing cracks form a similar T-shaped strucutre [49]. The phenomenon can be explained by the maximum stress relief criterion and the crack extension criterion [50,51]. In general, cracks tend to expand in a direction perpendicular to the local maximum tensile stress. After crack formation, the internal tensile stress perpendicular to the plane of existing crack has been released and the direction of the new maximum tensile stress must be parallel to the plane of the existing crack. Therefore, the extension direction of secondary cracks originating on existing cracks is always perpendicular to existing cracks (Fig 17).

**Fig 17:**
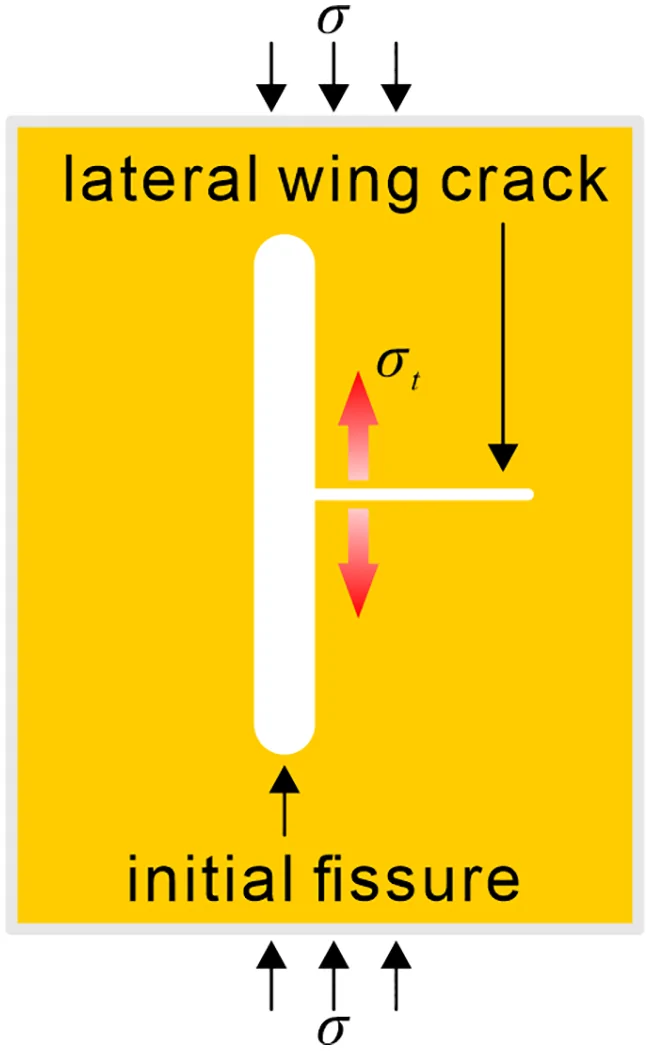
Extension wing cracks under compression.

The intensity of initial damage is influenced by the number of initial cracks. There is a mechanically weak zone between two initial cracks, which is highly susceptible to the formation of large volume damage cracks connecting the two initial cracks under loading. The number of large volume (1E + 12-1E + 13 μm^3^) damage cracks in the specimen TB reaches 1%, which is about 2–3 times higher than that of the single initial crack specimen. The location of the initial failure is related to the depth of the initial crack distribution. Shallow initial cracks mainly lead to damage in the middle, top or whole of the specimen, while deep initial cracks mainly lead to damage at the bottom of the specimen. When the spacing between the two initial cracks is large, the damage at the bottom of the specimen is dominated such that the initial failure of specimen TB is mainly concentrated in the region of the deep initial cracks. The initial failure morphology is related to the initial crack inclination angle. The initial failure morphology of 90°initial crack specimens are controlled by edge extension cracks, whereas the initial failure morphology of 0°and 45°initial crack specimens are controlled by edge wing cracks and lateral wing cracks. Differences in the types of secondary cracks formed at different extension angles resulted in diverse initial failure morphologies. These morphologies are influenced by the distribution depth and inclination of the millimeter-scale initial cracks and their combination characteristics.

The initial crack acts as a local structural defect. Under axial compression, the crack tip and crack side produce stress concentration. Depending on the crack inclination, the local stress field may be dominated by tensile opening, shear sliding, or mixed-mode deformation. Once the local stress intensity exceeds the local resistance of the loess matrix, secondary cracks initiate from the crack tip or crack side. For vertical initial cracks, the secondary cracks mainly appear as edge extension cracks, which extend approximately along the pre-existing crack direction. For horizontal and 45° inclined initial cracks, the local mixed tensile-shear promotes edge wing cracks and lateral wing cracks. For adjacent cracks, the interaction of stress fields between cracks creates a weak zone between the two cracks, thereby promoting crack interconnection.

On the other hand, the specimen size, crack continuity, and crack roughness may affect the soil failure modes. The crack evolution observed at the laboratory scale may differ from the cracking process at the field scale. Recent studies on defect-containing geomaterials have shown that crack propagation patterns may have significant scale effects, as the relative size relationships between the fracture zone, the material microstructure, and the specimen boundaries control the development of cracks [52,53]. Natural cracks commonly present as incomplete penetrations rather than ideal fully penetration. [54,55] found that the failure of cracked specimens is mainly affected by the initiation of cracks at the ends of existing cracks, the progressive fracturing of the intact rock bridge, and the formation of penetrating failure surfaces. The failure mode may transition between tensile failure, combined tensile-shear failure, shear failure, and pure shear failure depending on variations in crack continuity and crack combinations. Crack roughness also influences local mechanical behavior by altering crack surface contact, closure deformation, slide coherence, friction resistance, stress redistribution, and the development of secondary cracks. [56,57] indicate that rough cracks often fail to close completely and restrict coordinate sliding, thereby affecting crack propagation and the timing of failure. In natural shallow loess slopes, complex crack morphologies may lead to different failure modes. The applicability of the experimental observations in this study to shallow loess slopes at the field scale requires further verification and evaluation.

## Conclusion

In this paper, an experimental study of the initial failure state was carried out on shallow loess specimens containing millimeter-scale initial cracks with different characteristics. By quantitatively analyzing the spatial structure characteristics of the failure cracks under the initial failure state, the influence mechanism of the different characteristics of millimeter-scale initial cracks on the initial failure morphology (size, location and shape) is explored.

The experimental results show that specimens containing initial cracks are more easily to reach the initial failure state, and shallow cracks, 45° inclination cracks, and surface-shallow combined cracks reduce the axial stress by 78%, 65%, and 81%, respectively. The volume of failure cracks increased with the depth of the initial cracks, and the number of large volume cracks produced by deep initial cracks was twice as large as that of shallow initial cracks. The number of large volume cracks produced by 45° inclination cracks was 1.5 times and 2 times as large as that of horizontal and vertical cracks, respectively. Multiple initial cracks generate more large volume failure cracks than a single initial crack, with an increase in the number of cracks of about 98%.

This study obtained the spatial morphology of failure cracks in the initial failure state of original loess specimens containing millimeter-scale initial cracks by uniaxial compression coupled with CT real-time scanning experiments. This experimental method can provide a laboratory test reference for the study of damage process of surface or deep soil containing millimeter-scale initial cracks. However, due to uncertainties in the identification of initial failure points and CT segmentation along with limitations on the number of test specimens, the results of this study require comparison with a larger sample size to support broader statistical inferences. Further research will be carried out on the failure behavior of soils with millimeter-scale initial cracks under complex confining pressure, and aim to achieve conceptual advance in the relationship between millimeter-scale crack morphology and soil failure modes. The hydrological coupling, natural crack roughness, crack-size variability, repeated testing, and slope-scale boundary conditions should be considered to extend the present findings to real shallow loess hazards.

## Supporting information

S1 File Spatial geometric characteristic parameters of cracks. (XLSX)

S2 File Stress-strain curves. (XLSX)
